# The effects of somatostatin analogue therapy on pituitary tumor volume in patients with acromegaly

**DOI:** 10.1007/s11102-015-0677-y

**Published:** 2015-08-20

**Authors:** Annamaria Colao, Renata S. Auriemma, Rosario Pivonello

**Affiliations:** Dipartimento di Medicina Clinica e Chirurgia, Sezione di Endocrinologia, Università Federico II di Napoli, via S Pansini 5, 80131 Naples, Italy

**Keywords:** Acromegaly, Somatostatin analogue, Octreotide, Lanreotide, Pasireotide, Tumor volume

## Abstract

**Introduction:**

In nearly all cases, acromegaly is caused by excess GH from a pituitary adenoma, resulting in elevated circulating levels of GH and, subsequently, IGF-1. Treatment goals are to eliminate morbidity and restore the increased mortality to normal rates. Therapeutic strategies aim to minimize tumor mass and normalize GH and IGF-1 levels. Somatostatin analogues are the medical treatment of choice in acromegaly, as first-line or post-surgical therapy, and have proven efficacy in pituitary tumor volume reduction (TVR).

**Methods:**

Here we review the effects of somatostatin analogue therapy on pituitary tumor volume in patients with acromegaly.

**Results:**

TVR with somatostatin analogues may be mediated by direct anti-proliferative effects via activation of somatostatin receptors, or by indirect effects, such as angiogenesis inhibition, and is more pronounced when they are administered as first-line therapy. Various studies of first-line treatment with octreotide LAR have shown significant TVR in ≥73 % of patients. First-line treatment with lanreotide Autogel has shown evidence of TVR, although more studies are needed. In a recent randomized, double-blind, 12-month trial in 358 medical-treatment-naïve acromegaly patients, significant TVR was achieved by 81 % of patients administered pasireotide LAR and 77 % administered octreotide LAR. Pre-operative somatostatin analogue therapy may also induce TVR and improve post-operative disease control compared with surgery alone. TVR is progressive with prolonged treatment, and decreased IGF-1 levels may be its best predictor, followed by age and degree of GH decrease. However, TVR does not always correlate with degree of biochemical control.

**Conclusion:**

Somatostatin analogues (first- or second-line treatment) are the mainstay of medical therapy and, as first-line medical therapy, are associated with significant pituitary TVR in most patients.

## Introduction

The goals of treatment in acromegaly are to reduce morbidity and to restore the increased mortality to normal age- and sex-adjusted rates [1]. Therapeutic strategies are therefore aimed at removing tumor mass and/or stabilizing tumor growth while maintaining pituitary function, as well as normalizing the effects of growth hormone (GH) and insulin-like growth factor 1 (IGF-1) at target tissues [2]. Plasma levels of GH and/or IGF-1 are thereby controlled and any bulk effects of the tumor removed or minimized. At present, the main options for management of acromegaly are surgery and medical therapy. Radiotherapy may be considered when those options have failed. According to acromegaly treatment guidelines, choice of initial therapy should be determined by individual patient characteristics [3]. Surgery is still considered the first-line option for most patients, particularly when total tumor resection is feasible or relief of optic chiasm compression is needed, and when an experienced neurosurgeon is available, but some patients may be more suited to first-line medical therapy [2].

Surgical removal or debulking of the pituitary tumor is usually performed via the transsphenoidal route. The advantages of surgery include immediate lowering of GH and, subsequently, IGF-1 levels, elimination of the local mass effects of the tumor, and tissue sampling for analysis. The chances of surgical success are best in patients with a microadenoma (≤10 mm diameter), enclosed macroadenoma, and low pre-operative GH levels, and with highly experienced specialist neurosurgeons [4–6]. Advances in surgical instrumentation and imaging techniques have helped to improve outcomes. Nevertheless, there remain a number of potential complications of surgery, primarily hypopituitarism, but also diabetes insipidus (usually transient), meningitis, and cerebrospinal leaks [6]. Furthermore, only 40–70 % of patients who undergo surgical excision achieve normalization of GH and IGF-1 levels [4–6].

## Medical therapy

Since many patients have inadequately controlled disease following surgery, subsequent medical therapy and/or radiotherapy is often necessary. Although the use of medical therapy has traditionally been limited to the adjuvant setting, first-line treatment with pharmacological agents is appropriate in selected patients, including those with extrasellar tumors, patients at risk of complications of anesthesia, patients with severe complications of acromegaly, those who refuse to undergo surgery, and patients wishing to retain intact pituitary function [2, 3, 7–10]. Other patients may benefit from pre-operative medical therapy to reduce tumor size and improve clinical status before surgery, potentially improving outcomes [11].

### Somatostatin analogues

In healthy individuals, increased levels of GH stimulate the release of somatostatin, which then suppresses secretion of GH. In the presence of a GH-secreting pituitary tumor, plasma GH levels are excessive and the normal regulatory feedback loop fails. Cell-surface receptors for somatostatin have been identified in a variety of tissues, including the pituitary, and five subtypes have been characterized (designated sst_1–5_). Approximately 90 % of GH-secreting pituitary tumors express predominantly sst_2_ and sst_5_ [12]. The development of somatostatin analogues was a logical step towards the medical management of acromegaly. Compared with endogenous somatostatin, synthetic agents such as octreotide and lanreotide have much longer half-lives and are designed to bind selectively to sst_2_ and, to a lesser extent, sst_5_.

The first-generation somatostatin analogues, octreotide and lanreotide, are currently the medical treatment of choice in acromegaly, as both adjuvant and first-line therapy, and have demonstrated efficacy in controlling GH and IGF-1 levels and in reducing pituitary tumor volume. Octreotide and lanreotide are both available in long-acting depot formulations [octreotide long-acting repeatable (LAR) and lanreotide Autogel]. Pasireotide LAR is a novel, second-generation somatostatin analogue that has recently been shown to provide superior biochemical control versus octreotide LAR in medically naïve patients and versus continued treatment with octreotide LAR or lanreotide Autogel in inadequately controlled patients with acromegaly [13, 14]. The safety profile of pasireotide is similar to that of the first-generation somatostatin analogues, except for a higher frequency and degree of hyperglycemia [13, 14]. Pasireotide-induced hyperglycemia may be manageable with proactive monitoring and early intervention.

### Mechanisms of tumor volume reduction with somatostatin analogues

#### Direct effects of first-generation somatostatin analogues

The anti-proliferative effects of somatostatin analogues in pituitary adenomas may be mediated by somatostatin receptors, activation of which can induce apoptosis, cell cycle inhibition, and inhibition of growth factor effects [15, 16]. In cancer models, for example, it has been demonstrated that somatostatin analogues targeting sst_1_ and/or sst_2_ inhibit platelet-derived growth factor (PDGF)-stimulated ERK activity, with associated anti-proliferative effects [17]. Elsewhere, it was shown that sst_2_ receptors may also be involved in restoration of functional gap junctions (critical for maintenance of the differentiated state) by inducing expression of connexin [18]. The sst_2_ receptor has also been shown to exert anti-oncogenic properties. Buscail and colleagues demonstrated the loss of sst_2_ receptor expression in human pancreatic carcinoma and showed that restoration of the sst_2_ gene defect resulted in a significant reduction in cell growth and tumorigenicity [19]. Animal models have also shown that re-expression of sst_2_ resulted in decreased tumor growth [20, 21].

The underlying mechanism for these direct effects has not been fully elucidated, although certain pathways activated by binding to the sst_2_ receptor have a known role in mediating cell growth. Ligand interaction with sst_2_ initiates upregulation of protein tyrosine phosphatase (PTP), a key modulator of mitogenic effects that include cell differentiation and development. SHP-1, a negative regulator of hematopoietic cell signal transduction and negative regulator of cell signaling, is dissociated after treatment with somatostatin or octreotide, thereby dephosphorylating tyrosine kinase receptors [16, 22]. Additionally, sst_2_ activation has a role in modulating another central regulator of cell growth, the MAPK pathway, including phosphatidylinositol triphosphate kinases (PI3K) and Akt phosphorylation [23].

In a recent study using a pituitary tumor model including GH-secreting pituitary cells, through upregulation of the PI3K/Akt pathway, as well as mitogen-activated protein kinase pathways, octreotide increased both transcription of the mixed lineage leukemia (*MLL*) gene and levels of p27(Kip1), a protein that controls G1 cell cycle progression [24]. The authors concluded that the *MLL*–p27(Kip1) pathway may be a novel therapeutic target in pituitary tumors [24]. Additionally, a recent study evaluated the anti-proliferative effect of octreotide in combination with an mTOR inhibitor in pituitary tumor cells, as Akt activation reduces sensitivity to rapamycin and its analogues and octreotide acts as an upstream inhibitor of the PI3K/Akt pathway [25]. The study found that octreotide decreased levels of activated Ser(473)-phosphorylated Akt via modulating SHP-1, which, in combination with rapamycin, led to increased levels of p27(Kip1), as well as to macroscopic effects such as G1 cell cycle arrest [25].

In a pituitary cell model, it was shown that octreotide exerts its anti-proliferative action by increasing expression of the tumor suppressor gene *Zac1* [26]. Zac1 is a recently discovered novel zinc finger protein expressed in the pituitary gland and brain that induces cell cycle arrest and apoptosis [27, 28]. Octreotide was found to increase Zac1 levels by inhibiting the PI3K/Akt protein survival pathway, thereby preventing phosphorylation of Zac1 [26]. The same investigators subsequently demonstrated an association between pituitary tumor Zac1 expression and response to somatostatin analogue therapy in patients with acromegaly [29].

#### Indirect effects of first-generation somatostatin analogues

Somatostatin analogues may also act indirectly by inhibiting the release of growth factors and trophic hormones (such as IGF-1 and insulin), or through inhibition of angiogenesis, which limits tumor growth [15]. There is also evidence that downregulation of vascular endothelial growth factor (VEGF) may be how octreotide inhibits angiogenesis in pituitary tumors [30]. In neuroendocrine tumors, administration of octreotide significantly reduces VEGF secretion (likely via the PI3K/Akt pathway) [31]. Clinically, the anti-angiogenic effect of octreotide has been demonstrated in a small study of five patients with acromegaly, who showed a significant reduction in the functional vascularity of their pituitary tumors after 24 weeks of octreotide as first-line therapy [32].

#### Antiproliferative effects of pasireotide

Somatostatin analogues with different receptor binding profiles may also exert varying effects on cell growth. For example, pasireotide, the multireceptor-targeted somatostatin analogue, has approximately 30-, 11-, and 158-fold higher functional activity than octreotide on sst_1_, sst_3_, and sst_5_, respectively, and seven-fold lower activity on sst_2_ [33, 34]. Recent studies have shown that octreotide and pasireotide stimulate distinct patterns of sst_2A_ phosphorylation, with both compounds internalizing the receptor upon binding, but with pasireotide forming less stable beta-arrestin–sst_2A_ complexes compared with octreotide, leading to earlier recycling of sst_2A_ on the cell membrane [35]. Additionally, although an adenylyl cyclase inhibitor like somatostatin, pasireotide has an antagonistic effect on intracellular calcium stimulation and ERK phosphorylation [36]. A previous study of pituitary tumors had suggested that downregulation of phospho-ERK (and upregulation of p27) is associated with sst-mediated growth inhibition and that broader-spectrum somatostatin analogues are likely to play an increasing role in tumor types in which the MAPK pathway is over-expressed [37]. As a recent immunohistochemical study showed that different types of pituitary adenomas express a variety of sst, and that in tumors isolated from patients with acromegaly, sst_5_ and sst_1_ were more prevalent than sst_2A_, the authors concluded that multireceptor somatostatin analogues may be a useful approach, especially in somatotroph adenomas [38].

### Adjuvant therapy with somatostatin analogues

A retrospective study of 86 patients showed that debulking of tumors in patients poorly responsive to first-line therapy with somatostatin analogues enhanced the success rate in terms of achieving normal IGF-1 levels with post-surgical subcutaneous (sc) octreotide, octreotide LAR, or lanreotide Autogel [39]. All patients were treated with somatostatin analogues before and after surgery for at least 6 months. After the first course of treatment, pre-surgical magnetic resonance imaging (MRI) showed no change in tumor size in 49 %, mild volume reduction in 34 %, and moderate to notable volume reduction in 13 % of patients; four patients (5 %) had an increase in tumor size. After surgery, a decrease in tumor size of >75 % was noted in 50 (58 %) patients, of 50.1–75 % in 21 (24 %), of 25.1–50 % in 10 (12 %), and of <25 % in five (6 %). The success rate of post-surgical somatostatin analogue therapy, in terms of normalized IGF-1 and reduced GH levels, correlated with the amount of tumor removed surgically. There was no change in the cumulative prevalence of pituitary deficiency during the study [39].

In a meta-analysis of published studies enrolling patients with acromegaly to receive long-acting somatostatin analogues for at least 3 months’ duration, adjuvant treatment with octreotide LAR achieved GH levels <2.5 μg/L in 57 % of patients and normal IGF-1 in 67 % [40]. After a mean [standard deviation (SD)] treatment duration of 17.9 (1.5) months for all patients included in the meta-analysis, the percentage achieving >10 % tumor volume reduction was significantly higher with adjuvant octreotide LAR than with adjuvant lanreotide sustained release (SR; 47 vs 21 %, *P* < 0.0001) [40].

Long-term (40 months) adjuvant therapy with octreotide LAR has been shown to reduce GH and IGF-1 levels, and reduce tumor volume, in patients with persistent and poorly controlled acromegaly after transsphenoidal surgery, adjuvant radiation, and/or dopamine agonists, but without prior treatment with somatostatin analogues [41]. In this study, 33 patients were treated with octreotide LAR 20 mg every 28 days for 3 months, followed by individualized dose titration to achieve adequate control of IGF-1 and GH levels. Twenty-six patients were evaluable for tumor volume reduction at the 40-month time point. Tumor volume fell from a median [interquartile range (IQR)] of 1.18 (0.08–3.50) mL at baseline to 0.21 (0–2.1) mL at 40 months (*P* = 0.08), as measured by MRI.

In a review of five studies, including 79 patients treated with octreotide LAR as adjuvant therapy, 22 patients (28 %) achieved significant (>20 or >25 %) tumor volume reduction [42]. Octreotide LAR dosages in these studies ranged from 10 to 40 mg every 28 days, over a duration of 6–30 months.

In the same review, two studies showed that lanreotide SR adjuvant therapy achieved >20 % tumor volume reduction in eight of 87 (9 %) patients [42]. These included 3/3 patients treated with lanreotide SR 60 mg every 21–28 days for 6 months, but only 5/84 (6 %) patients treated with lanreotide SR 30 mg every 10–14 days for 24 months. A recent systematic review reported tumor volume reduction in 9–42 % of patients treated with lanreotide SR who had previously undergone surgery, radiotherapy, or treatment with drugs other than lanreotide [43].

Data from two recent studies showed a positive correlation between sst_2_ receptor expression in pituitary adenomas and both degree of tumor volume reduction and biochemical response with octreotide LAR adjuvant therapy [44, 45]. This may help to identify patients most likely to respond to first-generation somatostatin analogues, having failed to achieve adequate control with surgery alone.

### First-line therapy with somatostatin analogues

An overview of published studies evaluating somatostatin analogues as first-line therapy showed that 6–24 months of octreotide LAR therapy achieved significant (>20–30 %) tumor volume reduction in 73–85 % of patients, with an overall mean reduction in tumor volume of 35–68 % (Table 1) [13, 46–54].

**Table 1:** Summary of results from published studies of octreotide LAR, lanreotide SR, lanreotide Autogel, or pasireotide LAR as first-line therapy in acromegaly

Reference	No. of patients enrolled	Duration of treatment	Patients meeting criterion for GH control (%)	Patients with IGF-1 normalization (%)	Mean tumor volume reduction (%)	Patients with significant tumor volume reduction (definition of significant) (%)
Octreotide LAR						
Colao et al. [46]	15	12–24 months	73	53	53	80 (>20 %)
Ayuk et al. [47]	25	48 weeks	62	64	NR	NR
Jallad et al. [48]	28	6–24 months	NR	43	NR	76 (>25 %)
Colao et al. [49]	34	6 months	61	45.5	54^a^	74 (>30 %)
Cozzi et al. [50]	67	6–108 months	69	70	62	82 (>25 %)
Mercado et al. [51]	68	48 weeks	44	34	39	75 (>20 %)
Colao et al. [52]	56	24 months	86	84	68	NR
Colao et al. [53]	67	12 months	52	58	49	85 (>25 %)
Colao et al. [54]	40	48 weeks	NR	NR	35	73 (>20 %)
Colao et al. [13]	182	12 months	52	24	38	77 (≥20 %)
Lanreotide SR						
Baldelli et al. [56]	23	24 months	78	70	NR	22 (>20 %)
Attanasio et al. [57]	30	6–48 months	63	65	NR	50 (>25 %)
Lucas et al. [58]	104	1–>3 months		25	NR	29 (>20 %)
Lanreotide Autogel						
Colao et al. [59]	26	12 months	58	58	48	77 (>25 %)
Annamalai et al. [60]	30	6 months	60	40	39^a^	79 (≥20 %)
Caron et al. [61]	90	48 weeks	78	50	27	63 (≥20 %)
Pasireotide LAR						
Colao et al. [13]	176	12 months	48	39	40	81 (≥20 %)
Gadelha et al. [14]	130	6 months	35/43^b^	25/26^b^	14/10^b^	19/11^b^ (>25 %)

*NR* not reported

^a^Median tumor volume reduction

^b^Data shown for 40/60 mg doses

**Fig. 1:**
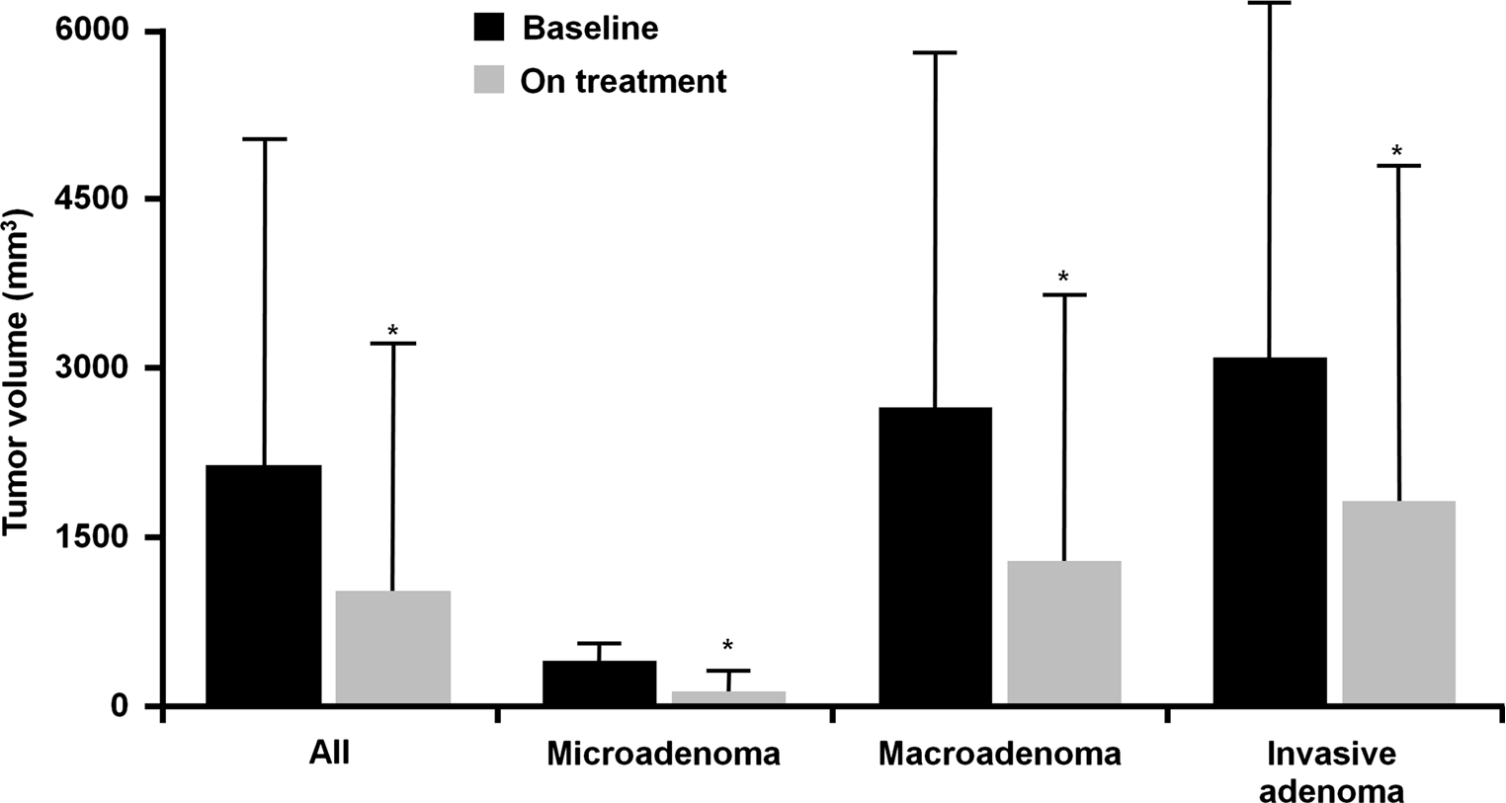
Tumor size before and during first-line octreotide LAR treatment, evaluated in the whole series and according to tumor type [50]. Republished with permission of The Endocrine Society, from Cozzi et al. [50]; permission conveyed through Copyright Clearance Center, Inc. *P < 0.05

The beneficial effects of long-term octreotide LAR treatment on tumor control were demonstrated in a study in which the drug was administered as first-line therapy for a median follow-up period of 48 months (range 6–108) [50]. Sixty-seven patients with acromegaly were enrolled in the study and started on octreotide LAR 20 mg every 28 days for 3 months, followed by individually titrated therapy. Overall, 82 % of patients achieved >25 % reduction in tumor volume, 44 % had >75 % reduction in tumor volume, and three patients had complete disappearance of their tumor [50]. Overall, mean (SD) tumor volume decreased significantly, from 2101 (2912) mm^3^ to 1010 (2196) mm^3^ (*P* < 0.0001), and none of the patients had any progression of tumor growth. The effects of octreotide LAR on tumor volume reduction were similar in patients with microadenomas, macroadenomas, and invasive adenomas (Fig. 1).

However, even a shorter duration of octreotide LAR as first-line therapy can be effective for reducing pituitary tumor volume. In a prospective, multicenter study of 98 patients treated with octreotide LAR 10–30 mg every 4 weeks, >20 % tumor volume reduction was reported in 63 % of patients after 24 weeks, and in 75 % at 48 weeks [51]. In this study, the greatest reductions in volume were observed with microadenomas. Furthermore, in a retrospective study of 67 patients, tumor volume reduction was observed as early as 3 months after starting octreotide LAR therapy, with a mean (SD) overall tumor volume reduction of 25.9 % (18.5 %) [53]. A significantly greater percentage of patients with microadenomas or enclosed macroadenomas achieved >25 % tumor volume reduction at 3 months than those with extrasellar and invasive macroadenomas (72.7 vs 35.6 %, *P* = 0.0009).

**Fig. 2:**
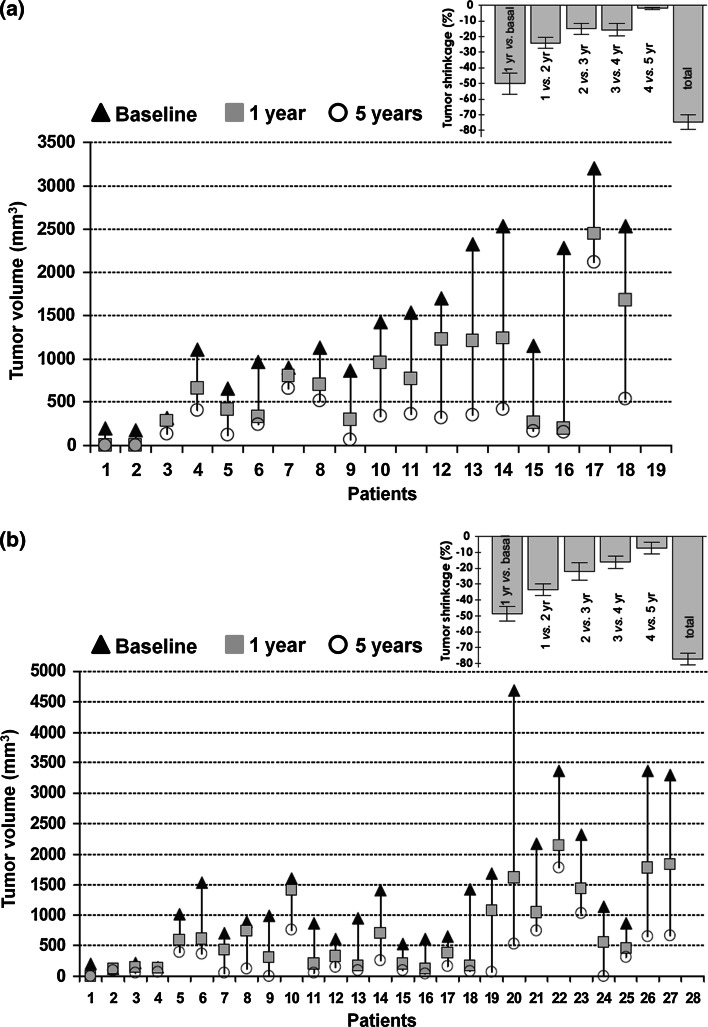
Progressive tumor volume changes with octreotide LAR in **a** women and **b** men. The *inset* graphs indicate percentage tumor volume reduction on a yearly basis and total volume change after 5 years [55]. Republished with permission of The Endocrine Society, from Colao et al. [55]; permission conveyed through Copyright Clearance Center, Inc.

It has also been shown that tumor shrinkage progresses with time (Fig. 2), even if, after 3 years of continuous treatment, only minimal further effects on tumor volume were seen [55]. The largest decreases in tumor volume generally occurred in the first year of treatment.

Compared with octreotide LAR, there are less data on lanreotide SR [56–58] or Autogel [59–61] on tumor volume reduction in the primary therapy setting. First-line therapy with lanreotide SR for 1–48 months was reported to achieve significant (>20–25 %) tumor volume reduction in 22–50 % of patients, whereas a 1-year study in which 26 newly diagnosed patients were treated with titrated lanreotide Autogel for 12 months reported >25 % tumor volume reduction in 77 % of patients, with a mean reduction in tumor volume of 48 % (Table 1). In a recent multicenter study of lanreotide Autogel 120 mg, 63 % of 90 patients had tumor volume reduction ≥20 % at 48 weeks (or the last available post-baseline value); 54 % had achieved clinically significant tumor volume reduction by week 12 [61]. A systematic review of the effects of lanreotide SR and Autogel on tumor mass showed that tumor volume reduction occurred more frequently among patients who received lanreotide as first-line therapy than in patients who had already been treated with surgery, radiotherapy, or other drugs [43].

### Predictors of tumor volume reduction with first-line somatostatin analogues

In the retrospective study by Colao et al. described above [53], the investigators found that tumor volume reduction at 12 months was predicted by both decrease in GH level and tumor volume reduction at 3 months, as long as octreotide LAR was titrated according to individual requirements. The percentage of patients with >25 % tumor volume reduction increased significantly from months 3 to 12 in those with extrasellar and invasive macroadenomas (from 35.6 % at month 3 to 82.2 % at month 12, *P* < 0.0001), as well as in those with microadenomas or enclosed macroadenomas (from 72.7 % at month 3 to 90.0 % at 12 months, *P* = 0.24). In this analysis, no correlation was found between tumor volume reduction and gender, age, baseline GH levels, or baseline tumor volume. Conversely, a prospective study in 99 patients showed that primary therapy with depot somatostatin analogues was associated with different degrees of tumor shrinkage in 75.5 % of patients with acromegaly [62]. IGF-1 levels after treatment were the best predictors of tumor shrinkage, followed by GH levels and age [62].

In the long-term study by Cozzi et al. [50] in which patients received octreotide LAR for up to 9 years, the greatest tumor volume reduction was observed in patients with higher baseline GH values, in those with the greatest changes in GH and IGF-1 during treatment, and in patients with macroadenomas (versus microadenomas; 81 vs 53 %, *P* = 0.0196).

A review of data from published studies of all the somatostatin analogues suggested that tumor volume reduction is progressive with prolonged treatment, and that decreased IGF-1 levels are the best predictor of tumor volume reduction, followed by age and degree of GH decrease [63]. Elsewhere, however, it has been observed that tumor volume reduction does not necessarily correlate with the degree of biochemical control [2, 42, 43]. As reported in a review discussing resistance to somatostatin analogues in patients with acromegaly, the concepts of ‘biochemical resistance’ and ‘tumor resistance’ (volume increase or <20 % volume reduction compared with baseline) should both be considered before a patient is determined to be unresponsive to somatostatin analogues, as significant tumor volume reduction has been achieved in the absence of complete biochemical control in certain patients [64]. The authors of a different recent review suggested that this dissociation may be explained by the hypothesized direct effects of somatostatin analogues on tumor tissue, the different mechanisms underlying their anti-mitotic and anti-secretory effects, and indirect effects such as anti-angiogenesis [43]. Indeed, the receptor signaling pathways that mediate the anti-proliferative effects of somatostatin analogues are usually different from those involved in the anti-secretory effects of these agents.

Furthermore, the relative prevalence of different receptor subtypes on the pituitary adenoma may affect outcome. In one case report, a patient with a large intra- and extrasellar macroadenoma was found to have a 50 % reduction in tumor size after 5 months of octreotide LAR, despite a failure to normalize IGF-1 and GH levels [65]. Subsequent resection and analysis of the tumor tissue revealed higher expression of the sst_5_ receptor compared with sst_2_, which the authors suggested may account for the lack of biochemical effect. Further studies are needed to clarify the precise effects of somatostatin analogue therapy in different patients.

### Pre-operative somatostatin analogue therapy

In general, pre-operative therapy with somatostatin analogues has been shown to have a beneficial effect on tumor size. For example, two non-randomized studies performed in the 1990s showed that the use of pre-operative octreotide achieved tumor volume reduction and softening of the tumor, facilitating subsequent tumor removal by surgery [66, 67]. In the first of these studies [66], octreotide was administered to 22 patients at doses of 150–600 μg/day for 3–6 months before surgery. Significant tumor volume reduction (≥30 %) was documented in five patients, and all 22 had significant reductions in GH and IGF-1 levels [66]. In the second study [67], 64 patients received octreotide 300–1500 μg/day for periods ranging from 3 to 9 weeks (n = 14, group 1) and from 3 to 39 months (n = 50, group 2). The investigators reported tumor volume reduction in 60 % of patients within 3 weeks, which was nearly maximal by 3–4 months. A greater number of patients in group 2 achieved >25 % tumor volume reduction (14 of 48 evaluable vs 1 of 14 in group 1). In both studies, pre-treatment with octreotide also improved the clinical status of patients prior to surgery and resulted in significantly better post-operative biochemical control compared with patients not pre-treated. A third uncontrolled study later performed in 90 patients with acromegaly found that pre-treatment with octreotide sc (mean daily dose 221 μg) for at least 3 months slightly improved the rate of biochemical control in patients with an invasive but potentially resectable macroadenoma [68]. During pre-treatment with octreotide sc, 31 % of patients achieved an MRI-confirmed reduction in tumor volume. Additionally, patients who received pre-operative treatment with octreotide sc were more likely to present to surgery with pituitary adenomas fluid or soft in texture, as well as white or gray in color, compared with patients who did not receive pre-operative octreotide [68].

In contrast, a small, randomized, controlled study in which octreotide was administered at a mean (SD) daily dosage of 470 (160) μg for a mean (SD) duration of 16.5 (10) weeks found that a non-significant reduction in tumor volume was reported during pre-treatment [69].

The Pre-operative Octreotide Treatment of Acromegaly (POTA) study was the first prospective, randomized trial comparing the outcome of 6 months of octreotide LAR pre-treatment with that of surgery alone [70]. The overall results showed that post-surgical normalization of IGF-1 was achieved in 45 % of pre-treated patients, compared with 23 % of patients who underwent direct surgery (*P* = 0.11) [70]. Among patients with macroadenomas, the difference in IGF-1 normalization was significantly in favor of the group with octreotide pre-treatment (50 vs 16 %, *P* = 0.017). In contrast to previous studies, there was no evidence of tumor softening with pre-operative octreotide LAR; in fact, the investigators noted that tumor firmness was common in this group. Furthermore, tumor volume change during pre-treatment was similar in both cured and non-cured patients post-surgery. Further studies are needed to clarify the effects of pre-operative octreotide LAR on tumor volume and consistency.

Two studies have reported pre-operative use of lanreotide SR 30 mg for up to 3 months [58, 71]. In one prospective, open-label study of 104 newly diagnosed acromegalic patients, lanreotide SR resulted in at least some tumor volume reduction in 66 %, and >20 % reduction in 29 %, of patients [58]. In the other non-randomized study, of 82 patients, tumor volume decreased significantly during lanreotide SR therapy, from 5662 to 2326 mm^3^ (*P* < 0.0001) [71]. This study also reported softening of the tumor, making it easier to remove [71].

An article that reviewed all the published literature on pre-operative somatostatin analogue therapy concluded that it should be considered in all patients with GH-secreting macroadenomas (including invasive) when the overall surgical remission rate for macroadenomas at the treating center is <50 % [72]. When deemed appropriate, somatostatin analogues should be given for 3–6 months before surgery. Patients with minimally invasive macroadenomas are most likely to benefit in terms of improved surgical remission.

### Pegvisomant

At present, pegvisomant is the only clinically available GH receptor antagonist for the treatment of acromegaly. Rather than inhibiting GH release, pegvisomant acts at the periphery to block the effects of GH on target tissues. By binding to GH receptors in the hepatocytes, pegvisomant blocks IGF-1 production. Pegvisomant is currently indicated for the treatment of patients who have had an inadequate response to surgery, radiotherapy, and/or prior medical therapy, or for those in whom such therapies are considered inappropriate. It is effective at normalizing IGF-1 levels (67.5 % after 5 years of treatment [73]), and it may also improve insulin resistance and cardiovascular risk parameters [74] and normalize elevated markers of bone turnover [75].

In contrast to somatostatin analogues, pegvisomant has not been shown to have an effect on tumor volume reduction. In a small proportion of cases, pituitary tumors have been shown to increase in size, including 30 (3.2 %) of 936 patients registered in the ACROSTUDY database with at least two MRI readings [76], and regular monitoring is therefore recommended. In patients previously treated with somatostatin analogues, it is possible that discontinuing somatostatin analogues increases the risk of tumor growth by removing the tumor-suppressive effects of somatostatin analogue therapy. The addition of pegvisomant to somatostatin analogue therapy may allow further reductions in IGF-1 and may also reduce the risk of tumor volume increase [76–79].

### Future therapies

Pasireotide is a novel, multireceptor-targeted somatostatin analogue with high binding affinity for sst_1,2,3_ and sst_5_ that has been approved for the treatment of Cushing’s disease [80–82] and acromegaly [13, 14, 83, 84]. Based on the differences in binding affinity and functional activity of pasireotide and octreotide, it can be speculated that in cells and tissues that express somatostatin receptor subtypes other than sst_2_, pasireotide may have a stronger inhibitory effect on hormone secretion and tumor growth than octreotide [34, 85]. Results from a 3-month Phase II study in patients with de novo or persistent/recurrent acromegaly showed that 39 % of patients had >20 % reduction in pituitary tumor volume after 3 months of treatment with pasireotide, which increased to 54 % of patients after 6 months of treatment [83]. Moreover, it was demonstrated in a large, Phase III randomized trial in patients with medically naïve acromegaly that pasireotide LAR and octreotide LAR have a similar effect on tumor volume reduction, despite the fact that pasireotide LAR was superior to octreotide LAR in providing biochemical control (Table 1) [13]. After 12 months of treatment, 81 % of pasireotide LAR patients had ≥20 % reduction in tumor volume, compared with 77 % of octreotide LAR patients. A recent study evaluated pasireotide LAR in patients with long-standing, inadequately controlled acromegaly, all of whom had received octreotide LAR 30 mg or lanreotide Autogel 120 mg monotherapy for ≥6 months before screening [14]. After 24 weeks of treatment, a greater proportion of patients receiving pasireotide LAR 40 and 60 mg achieved tumor volume decrease of >25 % compared with patients who continued receiving octreotide LAR or lanreotide Autogel (18.5 and 10.8 vs 1.5 %, respectively).

## Limitations

There are limitations associated with measuring tumor volume reduction during somatostatin analogue therapy. For example, computed tomography and MRI performed before the early 1990s had poor image resolution compared with more modern imaging [42]. This may have led to inaccurate estimation of the extent of tumor volume reduction, which therefore means that it is difficult to compare results across studies. More recently, standard formulae [46, 56] or geometric approximations [86] have commonly been used to estimate tumor volume; however, these methods lack precision for larger, more irregularly shaped tumors. There are also potential problems associated with observer subjectivity [42]. Recent studies have used more rigorous methods to assess tumor volumes. For example, in the Phase III pasireotide study, tumor volume was calculated by hand-drawing around the tumor circumference in coronal cross-sections, multiplying the area by slice thickness, and summing the resulting volumes across all slices containing tumor. Intra-observer variability of the blinded central reader was assessed independently by a third-party organization, and the results were found to be consistent, reproducible and, in most cases, unaffected by complex tumor anatomy [13]. In the PRIMARYS study, tumor volumes were measured centrally by three neuroradiologists blinded to the chronology of patients’ scans through the use of pre-specified methods, including computer modeling of tumor volumes, to ensure consistent and unbiased measurements [61]. Along these lines, future studies assessing tumor volume reduction with somatostatin analogue therapy should use robust methodology and follow the practices established in more recent trials.

## Conclusions

Somatostatin analogues are associated with significant pituitary tumor volume reduction in the majority of patients when administered as first-line therapy, and the reduction in tumor volume is progressive with prolonged treatment. In addition, pre-operative therapy with somatostatin analogues may be beneficial in some cases. The extent of decrease in IGF-1 levels during treatment may be the best predictor of tumor volume reduction, followed by age and degree of GH decrease, although tumor volume reduction does not always correlate with the degree of biochemical control. In a minority of patients, significant tumor volume reduction can be achieved with somatostatin analogues in the absence of biochemical control.
